# The Role of Education and Intellectual Activity on Cognition

**DOI:** 10.1155/2012/416132

**Published:** 2012-08-09

**Authors:** Jeanine M. Parisi, George W. Rebok, Qian-Li Xue, Linda P. Fried, Teresa E. Seeman, Elizabeth K. Tanner, Tara L. Gruenewald, Kevin D. Frick, Michelle C. Carlson

**Affiliations:** ^1^Johns Hopkins Bloomberg School of Public Health, Baltimore, MD 21205, USA; ^2^Johns Hopkins Center on Aging and Health, Johns Hopkins University, Baltimore, MD 21205, USA; ^3^Johns Hopkins School of Medicine, Baltimore, MD 21205, USA; ^4^Mailman School of Public Health, Columbia University, New York, NY 10032, USA; ^5^David Geffen School of Medicine, University of California at Los Angeles, CA 90095, USA; ^6^Johns Hopkins School of Nursing, Baltimore, MD 21205, USA; ^7^Davis School of Gerontology, University of Southern California, Los Angeles, CA 90089, USA

## Abstract

Although educational attainment has been consistently related to cognition in adulthood, the mechanisms are still unclear. Early education, and other social learning experiences, may provide the skills, knowledge, and interest to pursue intellectual challenges across the life course. Therefore, cognition in adulthood might reflect continued engagement with cognitively complex environments. Using baseline data from the Baltimore Experience Corps Trial, multiple mediation models were applied to examine the combined and unique contributions of intellectual, social, physical, creative, and passive lifestyle activities on the relationship between education and cognition. Separate models were tested for each cognitive outcome (i.e., reading ability, processing speed, memory). With the exception of memory tasks, findings suggest that education-cognition relations are partially explained by frequent participation in intellectual activities. The association between education and cognition was not completely eliminated, however, suggesting that other factors may drive these associations.

## 1. The Role of Education and Intellectual ****Activity on Cognition

Cognitive enrichment early in life may account for some of the variation in cognitive ability in adulthood. Consistently, higher educational attainment is associated with greater levels of cognitive performance [1–5], as well as with a reduced risk of dementia and Alzheimer's disease [6–9]. Although the exact mechanisms are unclear, one possibility is that educational experiences provide the foundation for continued intellectual stimulation across the life course, resulting in improved cognitive functioning in late adulthood. Prior research findings contribute to the plausibility of this assumption. First, educational attainment is often associated with greater participation in various lifestyle activities [10, 11], especially those that are cognitively demanding [12–14]. Second, the beneficial effects of maintaining an engaged lifestyle have been demonstrated across several studies, even when activities are introduced later in life [15–19].

Education may cultivate the knowledge, skills, and ability necessary for continued participation in intellectually demanding activities (e.g., reading, taking courses) well into later adulthood. According to the engagement hypothesis, individuals who continuously place significant demands on their intellectual resources (i.e., through multiple and complex decisions, ill-defined problem solving) may maintain or even enhance cognitive potential [3, 20, 21]. Therefore, compared to other forms of lifestyle activity, greater participation in intellectually demanding activities may be especially beneficial for cognitive function [3, 10, 14, 20–24]. For instance, Hultsch et al. [10] found that individuals who more frequently participated in novel information processing were less likely to show cognitive declines over time. Likewise, Ghisletta and colleagues [25] found that activities such as reading a book and playing games were related to changes in perceptual speed, whereas other forms of engagement (e.g., physical, social, and religious activities) were not associated with such changes. Conversely, activities low in cognitive stimulation, such as watching television, have been related to an increased risk of cognitive impairment [26]. Moreover, activities low in cognitive demand may be more prevalent among those with lower educational attainment [27].

Although several studies have explored the independent contributions of education or activity on cognition, few studies have explored these factors in combination. The current study examines whether educational attainment and late-life activities contribute independently to cognitive performance or if education-cognition relations can be at least partly explained by participation in activities in adulthood. Specifically, our work is driven by the following assumptions: (1) education will be associated with cognition, such that individuals with higher levels of educational attainment will demonstrate better performance on cognitive measures; (2) older adults with higher levels of educational attainment will report being more active, especially in intellectually demanding activities; (3) intellectual activities will influence cognition, such that participation in intellectually demanding activities (as compared to other forms of activity) will be related to better cognitive performance, independent of education. Consequently, we expect that the association between education and cognition will be attenuated (not completely eliminated) once participation in a wide variety of lifestyle activities is considered [28, 29]. Further, we also expect that the effects will be greatest for intellectually demanding activities, as these may be more strongly associated with both education and cognition [1–5, 10, 12–14].

We wish to be clear that this cross-sectional, correlational research does not allow us to establish the temporal ordering among variables and acknowledge that reciprocal relationships could be possible. Therefore, we cannot draw any conclusions regarding causal relationships between education, activity and cognition as implied by our theoretically based model (i.e., education leads to greater participation in activities, which in turn, promotes cognition). However, in the absence of longitudinal data, establishing associations among education, activity, and cognition allows us to better conceptualize plausible mechanisms for the promotion of cognitive health in adulthood.

## 2. Methods

We report data collected as part of the Baltimore Experience Corps Trial (see Fried and colleagues [30] for detailed description of recruitment procedures and study design), a community-based volunteer program designed as a health promotion model for older adults while simultaneously addressing the academic needs of elementary school children [30, 31]. Briefly, in this program older adults (age 60 and older) are trained and placed into elementary school classrooms (kindergarten to third grade) to serve as mentors and tutors for young children. To be eligible to serve as a volunteer, individuals had to meet screening criteria for general cognitive status (as indicated by a score of greater than 23 on the Mini-Mental State Exam (MMSE)) [32] and perform at a sixth grade reading level or higher on a measure of functional literacy, the Wide Range Achievement Test (WRAT-4) [33]. The data we report are based on baseline scores for measures before participants (*N* = 702) were randomly assigned to one of two conditions: to the intervention (Experience Corps program) or to a low-activity control group.

### 2.1. Sample

For purposes of the present analyses, individuals were included (*n* = 675) if they completed the Lifestyle Activities Questionnaire (LAQ) [16] and cognitive measures as part of the baseline assessment. Participants were, on average, 67 years of age (SD = 5.95, range = 60–89 years), had 13.9 years of education (SD = 2.94, range = 6–22 years), and were predominantly female (84.6%) and African American (90.5%) (Table 1). In addition, participants reported their current health as excellent, very good, or good (89.1%) and displayed very low levels of depressive symptoms (as indicated by a score less than 5 on the 15-item Geriatric Depression Scale; M = 1.5 symptoms; SD = 1.7) [34]. We have reported elsewhere that individuals who did not complete the LAQ at baseline tended to be older, reported poorer health, and demonstrated lower cognitive performance (as measured by the MMSE and WRAT-4) (*P's* < 0.05). There were no differences in educational attainment between those who completed and those who did not complete the LAQ measure at baseline [35].

### 2.2. Measures

#### 2.2.1. Education

Educational attainment was defined as the self-reported number of years of formal education completed.

#### 2.2.2. Activity

Frequency of participation in a wide range of activities was assessed via the Lifestyle Activities Questionnaire [16]. Participants rated their typical frequency of participation in various daily activities (e.g., cooking, singing, gardening, listening to music, reading) over the past year. Responses were made on a 6-point scale (never/less often than once a month, once a month, 2 to 3 times a month, once a week, a few times a week, and every day).

For analyses, we classified lifestyle activities into five activity domains to examine the effects of participation in specific types of activity on education-cognition relations (Table 2; also see Parisi et al. [35] for detailed information on activity items and domains). These activity domains were theoretically based on a comprehensive review of the existing literature [10, 12, 14, 16, 22, 36, 37] and have been used in our previous research [35]. Specifically, individual activity items were classified into* intellectual* (6 items: reading a book, reading a newspaper, balancing checkbook, using a computer, crossword puzzles, taking courses/classes), *social* (7 items: discussing local or national issues, visiting, clubs/organizations, attending church/religious service, playing cards/games, going to movies, going to plays/concerts), *physical *(3 items: shopping, gardening, hunting/fishing/camping), *creative* (4 items: singing, playing an instrument, cooking, drawing or painting, sewing/mending/fixing things), and passive (4 items: watching TV, listening to music, listening to the radio (not music), looking at art) activity domains (Table 2). The frequency of participation was calculated within each activity domain by averaging frequency responses to individual activity items, with lower numbers reflecting less participation.

#### 2.2.3. Cognition

Measures were selected to assess global cognitive status (MMSE) [32] and several distinct cognitive processes, such as reading ability, processing speed, and memory performance. *Reading ability* was assessed using the reading subtest of the Wide Range Achievement Test, version 4 (WRAT-4) [33]. To complete this test, participants are asked to read aloud a list of 15 letters and 55 words increasing in difficulty level (from cat to terpsichorean). Higher scores reflect a greater number of correctly pronounced words.* Processing speed* was assessed by the pattern comparison task [38] in which participants are asked to make “same” or “different” judgments as quickly as possible (for 30 seconds) for sequences of pairs of patterns. Higher scores reflect a greater number of correct responses. *Memory performance* was assessed by the Rey Auditory Verbal Learning Test (AVLT) [39, 40], capturing both immediate and delayed recall. Using a word-list learning paradigm, participants are first presented with a 15-word list (List A) and asked to recall the list (this process is repeated for five trials). Next, participants are presented with one trial of a second 15-word list (List B; interference) and asked to recall the list. Finally, participants are asked to recall the words on the initial list (List A) after a 20-minute delay (delayed recall trial). For scoring, immediate recall reflects the sum of words recalled on trials 1 to 5 (on List A) and delayed recall reflects total number of words recalled after a 20-minute delay. For each of these outcome measures, higher scores reflect greater memory ability.

#### 2.2.4. Covariates

Final models adjusted for demographics (e.g., age, sex, ethnicity/race), household income (for the past 12 months), self-reported health status (5-point scale; 1 = poor to 5 = excellent), and depressive symptoms as measured by the Geriatric Depression Scale (15-item) [34].

### 2.3. Data Analysis

**Figure 1:**
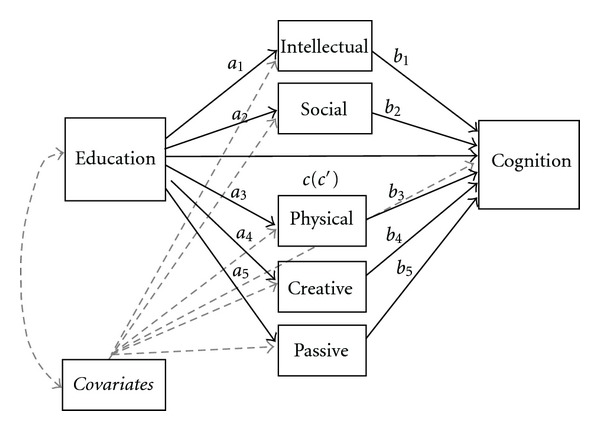
Example of multiple mediator model.

Multiple mediation models were applied to determine the following: (1) whether frequent participation in a wide range of lifestyle activities could partially explain the relationship between education and cognition, or (2) whether the relationship between education and cognition could be better explained by participation in specific activity domains (see Figure 1) [41, 42]. As we were interested in exploring the relative contributions of education and activities on distinct cognitive abilities, multiple mediation models were conducted separately for each cognitive outcome (e.g., reading ability, processing speed, memory; Figure 1). Analyses were conducted with IBM Statistical Package for the Social Sciences (SPSS) software, version 19 (see http://www.quantpsy.org/ for the SPSS macro command set for multiple mediation) [42].

Following the procedures defined by Preacher and Hayes [42], we examined both the *total *and *specific* indirect effects using 5,000 bootstrap samples to calculate the 95% bias-corrected and accelerated bootstrap confidence intervals (CI). These nonparametric bootstrapping techniques are often considered more statistically robust than traditional approaches (e.g., Sobel test, causal steps approach) because they do not assume normality in the sampling distribution [42–45]. The *total* indirect effect explains the *combined* contribution of activity domains (intellectual, social, physical, creative, passive) on education-cognition relations; whereas, the *specific *indirect effect tests the *unique *contribution of each activity domain, above and beyond participation in other domains. Point estimates were considered significant when zero was not included in the confidence interval.

## 3. Results

### 3.1. Correlations

As expected, greater educational attainment was related to better performance on cognitive measures (ranging from *r* = 0.09 for processing speed to *r* = 0.46 for reading ability), as well as with overall frequency of activity (*r* = 0.14) (Table 3). Educational attainment was also related to greater participation in intellectual and physical activities (*r* = 0.26 and 0.14, resp.). Additionally, greater participation in intellectual activities was consistently associated with performance on cognitive tasks (ranging from *r* = 0.11 for delayed memory recall to *r* = 0.22 for reading ability). 

### 3.2. Multiple Mediation Models

The results from the bootstrapping analyses showed that the total indirect effect (i.e., aggregate effect of participation across the five activity domains) was significant for the measures of global cognition (point estimate = 0.120; CI_.95_ = 0.000, 0.025), reading ability (point estimate = 0.062; CI_.95_ = 0.014, 0.120), and processing speed (point estimate = 0.075; CI_.95_ = 0.033, 0.131) (Table 4). It is important to note that in each of these models, education-cognition relations were not completely eliminated (i.e., the association between education on cognition remained significant). These findings indicate that, taken as a set, frequent participation in a wide range of lifestyle activities partially accounts for the effects of education on these cognitive abilities. Further inspection of the specific indirect effects (e.g., a_1_b_1_ versus a_2_b_2_ in Figure 1) indicated that this effect only held true for intellectually challenging activities (point estimate = 0.014; CI_.95_ = 0.000, 0.025 for global cognition; point estimate = 0.070; CI_.95_ = 0.027, 0.125 for reading ability; point estimate = 0.078; CI_.95_ = 0.040, 0.134 for processing speed; Table 4), controlling for all other activity domains. Thus, these findings suggest that greater participation in social, physical, creative, or passive activity did not contribute to the total indirect effect above and beyond participation in intellectual activity for measures of global cognition, reading ability, and processing speed. We did not find similar associations (neither the total nor specific indirect effects were significant) for the AVLT immediate or delayed memory task.

## 4. Discussion

The goal of the present study was to examine whether participation in a wide range of activities (intellectual, social, physical, creative, and passive) could account for the relationship between education and cognition in adulthood. Generally, our findings suggest that education-cognition relations can be partially explained by frequent participation in intellectual demanding activities [19, 29, 46].

As suggested earlier, educational experiences may provide the necessary knowledge, understanding, skills, and competencies for establishing a lifetime of participation in cognitive challenges. In fact, individuals with higher levels of educational attainment tend to allocate more time and put forth more effort when engaging in intellectually complex activities [47]. As a result, the accumulated exposure to cognitively charged environments may have a direct beneficial effect on brain structure and function, resulting in greater neurological development (e.g., increase in synaptic density) or more efficient use of existing brain networks [48–50]. In addition to such neuroprotective effects, continued practice of cognitive skills may develop compensatory strategies to help maintain cognition in the face of age-related declines [51] or may bolster perceptions of confidence and competence in one's skills and abilities, potentially leading to more frequent engagement in cognitively demanding environments [36, 52].

It is important to mention, however, that the association between education and cognition was not completely eliminated by participation in intellectually demanding activities, suggesting that both educational attainment and intellectual endeavors may independently benefit late-life cognitive performance [29]. In other words, although education may provide a foundation for continued engagement in intellectually demanding environments across the life course, it is also the “choices we make, not chance, that determines our fate” [53]. 

The few studies that have previously investigated these hypothesized pathways have yielded mixed findings. Similar to our findings, Kleigel and colleagues [29] also demonstrated the importance of both education and intellectual stimulation for cognitive performance among the oldest old. A more recent study by Soubelet [54], however, failed to find such associations. The discrepancies may be attributable, in part, to differences in the selected sample, exclusively among centenarians (M = 100.21 years; SD = 0.40) [29] or across a large range of ages (18–96 years) [54]. Further, the definition of intellectual activity differed across studies, potentially impacting the significance of findings. For instance, some of the intellectual activities used in the study by Soubelet [54] were included in our social domain (e.g., theater, cinema, religious participation), for which we did not find significant associations. It should also be noted that the few reports examining whether education-cognition associations could be explained by intellectual activities did not simultaneously consider participation in other forms of engagement. In fact, there is very little work that has examined the relations between education and the types of lifestyle activities that were measured in the current study. The application of multiple mediation models allowed us to test the effects of participation in a wide range of activities (*total indirect* effect), as well as independent associations with specific activity domains (*specific indirect* effect), on cognitive performance. To our knowledge, no other study has explored whether participation in various forms of activity can potentially explain the association between education and cognition.

Although our findings contribute to the relatively few studies that have examined education-cognition pathways, several limitations need to be addressed. First and foremost, as our data were cross-sectional, we were unable to distinguish whether variation in cognition resulted from age-related decline or from earlier life experiences. We also were unable to establish temporal precedence between activity and cognition. As such, it is also likely that cognitive ability could lead to greater activity [23, 55], rather than activity driving cognition as suggested in the current study. With this said, while correlation does not imply causation, establishing covariation among variables is a necessary (albeit insufficient) condition for causality. In the literature, several questions regarding the determinants and effects of an active lifestyle have yet to be answered [15]. Even though we cannot draw causal assumptions from cross-sectional data, our correlational findings help define what causal models are plausible. More longitudinal investigations are needed to test these competing models, as well as to determine how engaging in cognitively complex challenges across different periods of the lifespan impacts cognition in adulthood [21, 56]. 

We also acknowledge that investigating these associations within the context of a school-based, intensive volunteer program attracted a relatively healthy, active group of volunteers potentially limits the generalizability of our findings to other populations. However, individuals included in this study reported a high prevalence of chronic health conditions (e.g., diabetes, stroke, hypertension, and vascular disease) and comorbidity, placing them at a disproportionately greater risk for cognitive and physical impairments [57]. We also recognize that the *number of years* of education does not directly translate to the *quality *of these educational experiences [58, 59]. This is especially salient given that many of the individuals enrolled in the Baltimore Experience Corps Trial were educated prior to desegregation, a time when the quality of and access to education was not equal for African Americans. 

Lastly, our measure of activity (LAQ) was developed to capture participation in a broad range of lifestyle activities (e.g., cognitive, social, physical), with a limited number of items reflecting each activity domain. This is especially true for physical activities, which have demonstrated associations with both education and cognition in prior research [48]. However, these neurobiological findings have not translated as well to epidemiologic studies of dementia risk, where self-reported frequency of physical activity is the standard [60, 61]. For instance, Wang and colleagues [62] did not observe an association between physical activity and dementia incidence after accounting for participation in social, cognitive, and productive activities. Nonetheless, findings may have differed if more extensive activity measures were implemented. Further, education and lifestyle activities are not the only forms of experiential richness. For instance, occupational complexity has been consistently linked to cognition in later life [56, 63]. Moreover, occupational status is often associated with other factors (e.g., finances, time) that may impact selection and participation in activities over the life course [64]. Unfortunately, we did not have a reliable measure of occupational history or complexity and were unable to explore these relations in our dataset.

Consistent with the engagement hypothesis [3, 10], remaining actively engaged in activities may provide a protective mechanism against cognitive decline and dementia in later life. Although there was some evidence that education-cognition relations could be partially explained by greater participation in intellectual activities, both education and activities uniquely contributed to cognition in adulthood. As such, interventions such as the Experience Corps program which promote broad-based engagement may help older adults maintain, or potentially enhance, cognitive function. Further research is recommended to replicate these important findings with similar activities in varied populations. 

## Figures and Tables

**Table 1:** Demographic characteristics of sample.

	%	M	SD
Age (years)		67.49	5.95
Education (years)		13.85	2.94
Elementary school (K-8)	2.3		
High school/equivalent (9–12)	40.2		
College (13–16)	41.2		
Postgraduate	13.1		
Other	1.1		
Income (past 12 months)			
Less than $5,000	6.1		
$5,000–14,999	23.1		
S15,000–34,999	35.2		
$35,000–74,999	27.4		
$75,000 or greater	6.8		
Sex			
Male	15.4		
Female	84.6		
Race			
African American	90.5		
European American	5.1		

**Table 2:** Activity domains from the lifestyle activities questionnaire.

Activities	Percentage of individuals reporting activity	M	SD
Intellectual			
Reading a book	88.7	3.1	l.8
Reading a newspaper	87.7	3.6	1.7
Balancing checkbook	82.6	2.0	1.5
Using a computer	57.0	2.2	2.2
Crossword puzzles	49.0	1.6	2.0
Taking courses or classes	25.7	0.6	1.2
Social			
Discussing local or national issues	96.0	3.9	1.3
Attending church religious service	91.3	3.0	1.3
Visiting	87.2	2.3	1.4
Clubs/organizations	78.6	2.2	1.6
Playing cards or games	49.4	1.2	1.5
Going to movies	33.9	0.5	0.9
Going to plays concerts	33.5	0.4	0.7
Physical			
Shopping	98.8	3.3	1.0
Gardening	46.9	1.5	l.8
Hunting, Fishing, Camping	3.7	0.1	0.3
Creative			
Preparing food	97.6	4.2	1.1
Sewing, mending, fixing things	78.2	2.2	1.6
Singing, playing instrument	66.6	2.5	2.0
Drawing or Painting	21.0	0.5	1.1
Passive			
Watching TV	99.1	4.8	0.6
Listening to music	99.1	4.6	0.9
Listening to radio (not music)	89.3	3.8	1.6
Looking at art	58.9	1.4	1.6

Note: Averages based on 6-point scale; 1 = never, 6 = everyday.

**Table 3:** Unadjusted correlations among education, activity, and cognition.

	Cognition					
	Education	MMSE	WRAT-4	Speed	Memory: immediate	Memory: delayed
Education	1.00	0.17^∗∗^	0.46^∗∗^	0.09^∗^	0.10^∗∗^	0.11^∗∗^
Activity level						
Frequency, overall	0.14^∗∗^	0.08^∗^	0.05	0.15^∗∗^	0.12^∗∗^	0.10^∗^
Activity type						
Intellectual	0.26^∗∗^	0.12^∗∗^	0.22^∗∗^	0.16^∗∗^	0.14^∗∗^	0.11^∗∗^
Social	−0.04	−0.01	−0.13^∗∗^	0.08^∗^	0.05	0.03
Physical	0.14^∗∗^	−0.03	0.08^∗^	0.04	0.04	0.01
Creative	0.01	0.02	−0.06	0.06	0.12^∗∗^	0.10^∗^
Passive	0.07	−0.02	−0.02	0.03	−0.01	−0.01

Note: *P < 0.05. **P < 0.01. MMSE: Mini-Mental State Exam; WRAT: Wide Range Achievement Test-4.

**Table 4:** Indirect effects of education on cognition through activity.

			BCa 95% Cl	
Indirect effects	Estimate	SE	Lower	Upper
MMSE				
Total	.012	.006	.000	.025
Intellectual	.014	.006	.003	.027
Social	.000	.001	−.003	.003
Physical	−.002	.002	−.008	.002
Creative	−.001	.001	−.005	.001
Passive	−.001	.002	−.006	.001

WRAT-4				
Total	.062	.027	.014	.120
Intellectual	.070	.024	.027	.125
Social	.000	.004	−.010	.009
Physical	.003	.009	−.011	.027
Creative	−.004	.008	−.029	.005
Passive	−.007	.009	−.033	.004

Processing speed				
Total	.075	.025	.033	.131
Intellectual	.078	.024	.040	.134
Social	.000	.004	−.007	.007
Physical	.001	.008	−.016	.018
Creative	.001	.004	−.007	.012
Passive	−.004	.007	−.025	.003

Memory: immediate recall				
Total	.043	.033	−.015	.112
Intellectual	.050	.031	−.004	.119
Social	.002	.005	−.009	.012
Physical	−.004	.012	−.033	.017
Creative	.004	.010	−.006	.036
Passive	−.008	.010	−.041	.004

Memory: delayed recall				
Total	.005	.012	−.017	.030
Intellectual	.010	.012	−.012	.034
Social	.000	.002	−.005	.004
Physical	−.005	.005	−.018	.002
Creative	.001	.003	−.002	.012
Passive	−.001	.003	−.012	.002

Note: IV: independent variable (education, in years); DV: dependent variable (cognitive outcomes); BCa 95% Cl: bias-corrected and accelerated confidence intervals. Total indirect effect represents the sum of the indirect effects for specific activity pathways. All models were adjusted for age, sex, race, household income (past 12 months), health, and depression.

## References

[1] Albert MS, Jones K, Savage CR (1995). Predictors of cognitive change in older persons: MacArthur studies of successful aging. *Psychology and Aging*.

[2] Lyketsos CG, Chen LS, Anthony JC (1999). Cognitive decline in adulthood: an 11.5-year follow-up of the Baltimore Epidemiologic Catchment Area study. *American Journal of Psychiatry*.

[3] Schaie KW (2005). *Developmental Influences on Adult Intelligence: The Seattle Longitudinal Study*.

[4] Tucker-Drob EM, Johnson KE, Jones RN (2009). The cognitive reserve hypothesis: a longitudinal examination of age-associated declines in reasoning and processing speed. *Developmental Psychology*.

[5] Wilson RS, Hebert LE, Scherr PA, Barnes LL, De Leon CFM, Evans DA (2009). Educational attainment and cognitive decline in old age. *Neurology*.

[6] Bennett DA, Wilson RS, Schneider JA (2003). Education modifies the relation of AD pathology to level of cognitive function in older persons. *Neurology*.

[7] Carlson MC, Brandt J, Carson KA, Kawas CH (1998). Lack of relation between race and cognitive test performance in Alzheimer’s disease. *Neurology*.

[8] Sharp ES, Gatz M (2011). Relationship between education and dementia: an updated systematic review. *Alzheimer Disease & Associated Disorders*.

[9] Stern Y, Gurland B, Tatemichi TK, Ming Xin Tang, Wilder D, Mayeux R (1994). Influence of education and occupation on the incidence of Alzheimer’s disease. *Journal of the American Medical Association*.

[10] Hultsch DF, Hertzog C, Small BJ, Dixon RA (1999). Use it or lose it: engaged lifestyle as a buffer of cognitive decline in aging?. *Psychology and Aging*.

[11] Strain LA, Grabusic CC, Searle MS, Dunn NJ (2002). Continuing and ceasing leisure activities in later life: a longitudinal study. *Gerontologist*.

[12] Aartsen MJ, Smits CHM, Van Tilburg T, Knipscheer KCPM, Deeg DJH (2002). Activity in older adults: cause or consequence of cognitive functioning? A longitudinal study on everyday activities and cognitive performance in older adults. *Journals of Gerontology*.

[13] Lachman ME, Agrigoroaei S, Murphy C, Tun PA (2010). Frequent cognitive activity compensates for education differences in episodic memory. *American Journal of Geriatric Psychiatry*.

[14] Wilson RS, Bennett DA, Beckett LA (1999). Cognitive activity in older persons from a geographically defined population. *Journals of Gerontology*.

[15] Bielak AAM (2010). How can we not ’lose it’ if we still don’t understand how to ’use it’? unanswered questions about the influence of activity participation on cognitive performance in older age—a mini-review. *Gerontology*.

[16] Carlson MC, Parisi JM, Xia J (2011). Lifestyle activities and aging: variety may be the spice of life. *Journal of the International Neuropsychological Society*.

[17] Hertzog C, Kramer AF, Wilson RS, Lindenberger U (2008). Enrichment effects on adult cognitive development: can the functional capacity of older adults be preserved and enhanced?. *Psychological Science in the Public Interest, Supplement*.

[18] Stine-Morrow EAL, Parisi JM, Morrow DG, Park DC (2008). The effects of an engaged lifestyle on cognitive vitality: a field experiment. *Psychology and Aging*.

[19] Wilson RS, Barnes LL, Krueger KR, Hoganson G, Bienias JL, Bennett DA (2005). Early and late life cognitive activity and cognitive systems in old age. *Journal of the International Neuropsychological Society*.

[20] Lövdén M, Bäckman L, Lindenberger U, Schaefer S, Schmiedek F (2010). A theoretical framework for the study of adult cognitive plasticity. *Psychological Bulletin*.

[21] Schooler C, Mulatu MS (2001). The reciprocal effects of leisure time activities and intellectual functioning in older people: a longitudinal analysis. *Psychology and Aging*.

[22] Bielak AAM, Hughes TF, Small BJ, Dixon RA (2007). It’s never too late to engage in lifestyle activities: significant concurrent but not change relationships between lifestyle activities and cognitive speed. *Journals of Gerontology*.

[23] Small BJ, Dixon RA, McArdle JJ, Grimm KJ (2012). Do changes in lifestyle engagement moderate cognitive decline in normal aging? Evidence from the Victoria Longitudinal Study. *Neuropsychology*.

[24] Wilson RS, Barnes LL, Bennett DA (2003). Assessment of lifetime participation in cognitively stimulating activities. *Journal of Clinical and Experimental Neuropsychology*.

[25] Ghisletta P, Bickel JF, Lövdén M (2006). Does activity engagement protect against cognitive decline in old age? Methodological and analytical considerations. *Journals of Gerontology*.

[26] Wang JYJ, Zhou DHD, Li J (2006). Leisure activity and risk of cognitive impairment: the Chongqing aging study. *Neurology*.

[27] Wilson RS, Bennett DA, Gilley DW, Beckett LA, Barnes LL, Evans DA (2000). Premorbid reading activity and patterns of cognitive decline in Alzheimer disease. *Archives of Neurology*.

[28] Fritsch T, McClendon MJ, Smyth KA, Lerner AJ, Friedland RP, Larsen JD (2007). Cognitive functioning in healthy aging: the role of reserve and lifestyle factors early in life. *Gerontologist*.

[29] Kliegel M, Zimprich D, Rott C (2004). Life-long intellectual activities mediate the predictive effect of early education on cognitive impairment in centenarians: a retrospective study. *Aging and Mental Health*.

[30] Fried LP, Carlson MC, Freedman M (2004). A social model for health promotion for an aging population: initial evidence on the Experience Corps model. *Journal of Urban Health*.

[31] Rebok GW, Carlson MC, Glass TA (2004). Short-term impact of Experience Corps participation on children and schools: results from a pilot randomized trial. *Journal of Urban Health*.

[32] Folstein MF, Folstein SE, McHugh PR (1975). ‘Mini mental state’. A practical method for grading the cognitive state of patients for the clinician. *Journal of Psychiatric Research*.

[33] Wilkinson GS, Robertson GJ (2006). *Wide Range Achievement Test-Fourth Edition*.

[34] Sheikh JI, Yesavage JA (1986). Geriatric Depression Scale (GDS): recent evidence and development of a shorter version. *Clinical Gerontologist*.

[35] Parisi JM, Rebok GW, Seeman TE, Tanner EK, Tan E, Fried LP Lifestyle activities in sociodemographically at-risk urban, older adults prior to participation in the Baltimore Experience Corps trial.

[36] Jopp D, Hertzog C (2007). Activities, self-referent memory beliefs, and cognitive performance: evidence for direct and mediated relations. *Psychology and Aging*.

[37] Parisi JM, Stine-Morrow EA, Noh SR, Morrow DG (2009). Predispositional engagement, activity engagement, and cognition among older adults. *Neuropsychology, Development, and Cognition*.

[38] Salthouse TA, Babcock RL (1991). Decomposing adult age differences in working memory. *Developmental Psychology*.

[39] Rey A (1941). L'examen psychologique dans les cas d'encephalopathie tramatique. *Archives de Psychologie*.

[40] Schmidt M (2004). *Rey Auditory and Verbal Learning Test: A Handbook*.

[41] MacKinnon DP, Rose JS, Chassin L, Presson CC, Sherman SJ (2000). Contrasts in multiple mediator models. *Multivariate Applications in Substance Use Research: New Methods for New Questions*.

[42] Preacher KJ, Hayes AF (2008). Asymptotic and resampling strategies for assessing and comparing indirect effects in multiple mediator models. *Behavior Research Methods*.

[43] MacKinnon DP, Lockwood CM, Hoffman JM, West SG, Sheets V (2002). A comparison of methods to test mediation and other intervening variable effects. *Psychological Methods*.

[44] Shrout PE, Bolger N (2002). Mediation in experimental and nonexperimental studies: new procedures and recommendations. *Psychological Methods*.

[45] Williams J, MacKinnon DP (2008). Resampling and distribution of the product methods for testing indirect effects in complex models. *Structural Equation Modeling*.

[46] Arbuckle TY, Gold DP, Chaikelson JS, Lapidus S (1994). Measurement of activity in the elderly: the activities checklist. *Canadian Journal on Aging*.

[47] Parisi JM (2010). Engagement in adulthood: perceptions and participation in daily activities. *Activities, Adaptation and Aging*.

[48] Colcombe S, Kramer AF (2003). Fitness effects on the cognitive function of older adults: a meta-analytic study. *Psychological Science*.

[49] Park DC, Reuter-Lorenz P (2009). The adaptive brain: aging and neurocognitive scaffolding. *Annual Review of Psychology*.

[50] Scarmeas N, Stern Y (2004). Cognitive reserve: implications for diagnosis and prevention of Alzheimer’s disease. *Current Neurology and Neuroscience Reports*.

[51] Baltes PB, Baltes MM, Baltes PB, Baltes MM (1990). Selective optimization with compensation. *Successful Aging: Perspectives from the Behavioral Sciences*.

[52] Lachman ME (2006). Perceived control over aging-related declines: adaptive beliefs and behaviors. *Current Directions in Psychological Science*.

[53] Stine-Morrow EAL (2007). The dumbledore hypothesis of cognitive aging. *Current Directions in Psychological Science*.

[54] Soubelet A (2011). Engaging in cultural activities compensates for educational differences in cognitive abilities. *Aging, Neuropsychology, and Cognition*.

[55] Gow AJ, Corley J, Starr JM, Deary IJ (2012). Reverse causation in activity-cognitive ability associations: the Lothian Birth Cohort 1936. *Psychology and Aging*.

[56] Schooler C, Mulatu MS, Oates G (1999). The continuing effects of substantively complex work on the intellectual functioning of older workers. *Psychology and Aging*.

[57] Alzheimer’s Association (2010). *Alzheimer’s Disease Facts and Figures*.

[58] Manly JJ, Jacobs DM, Touradji P, Small SA, Stern Y (2002). Reading level attenuates differences in neuropsychological test performance between African American and White elders. *Journal of the International Neuropsychological Society*.

[59] Manly JJ, Touradji P, Tang MX, Stern Y (2003). Literacy and memory decline among ethnically diverse elders. *Journal of Clinical and Experimental Neuropsychology*.

[60] Podewils LJ, Guallar E, Kuller LH (2005). Physical activity, APOE genotype, and dementia risk: findings from the Cardiovascular Health Cognition Study. *American Journal of Epidemiology*.

[61] Verghese J, Lipton RB, Katz MJ (2003). Leisure activities and the risk of dementia in the elderly. *New England Journal of Medicine*.

[62] Wang HX, Karp A, Winblad B, Fratiglioni L (2002). Late-life engagement in social and leisure activities is associated with a decreased risk of dementia: a longitudinal study from the Kungsholmen Project. *American Journal of Epidemiology*.

[63] Andel R, Crowe M, Kareholt I, Wastesson J, Parker MG (2011). Indicators of job strain at midlife and cognitive functioning in advanced old age. *Journals of Gerontology*.

[64] Kramer AF, Bherer L, Colcombe SJ, Dong W, Greenough WT (2004). Environmental influences on cognitive and brain plasticity during aging. *Journals of Gerontology*.

